# Effect of Probiotics on Depression: A Systematic Review and Meta-Analysis of Randomized Controlled Trials

**DOI:** 10.3390/nu8080483

**Published:** 2016-08-06

**Authors:** Ruixue Huang, Ke Wang, Jianan Hu

**Affiliations:** Department of Occupational and Environmental health, Xiangya school of Public Health, Central South University, Changsha 410078, Hunan, China; huangruixue@csu.edu.cn (R.H.); wangke2138@126.com (K.W.)

**Keywords:** probiotics, depression, meta-analysis, randomized controlled trial

## Abstract

It has been reported that gut probiotics play a major role in the bidirectional communication between the gut and the brain. Probiotics may be essential to people with depression, which remains a global health challenge, as depression is a metabolic brain disorder. However, the efficacy of probiotics for depression is controversial. This study aimed to systematically review the existing evidence on the effect of probiotics-based interventions on depression. Randomized, controlled trials, identified through screening multiple databases and grey literature, were included in the meta-analysis. The meta-analysis was performed using Review Manager 5.3 software using a fixed-effects model. The meta-analysis showed that probiotics significantly decreased the depression scale score (MD (depressive disorder) = −0.30, 95% CI (−0.51–−0.09), *p* = 0.005) in the subjects. Probiotics had an effect on both the healthy population (MD = −0.25, 95% CI (−0.47–−0.03), *p* = 0.03) and patients with major depressive disorder (MDD) (MD = −0.73, 95% CI (−1.37–−0.09), *p* = 0.03). Probiotics had an effect on the population aged under 60 (MD = −0.43, 95% CI (−0.72–−0.13), *p* = 0.005), while it had no effect on people aged over 65 (MD = −0.18, 95% CI (−0.47–0.11), *p* = 0.22). This is the first systematic review and meta-analysis with the goal of determining the effect of probiotics on depression. We found that probiotics were associated with a significant reduction in depression, underscoring the need for additional research on this potential preventive strategy for depression.

## 1. Introduction

Depression is a common mental disorder, which can be long-lasting or recurrent, substantially impairing an individual’s ability to function in their daily life [1]. People with a depressed mood can feel sad, anxious, empty, hopeless, helpless, worthless, guilty, irritable, ashamed or restless [2]. They may lose interest in physical activities, have a loss of appetite or overeating, have problems concentrating, remembering details or making decisions and, even more seriously, may attempt or commit suicide. It is also increasingly recognized that sub-clinical levels of depressive symptoms are found in healthy populations [3]. Because of their impact and widespread prevalence, depressive symptoms are a growing public health concern. Nearly 20% of the population, at some point in their lifetime, will suffer depression [4]. Currently, there are 350 million people plagued by depression, and the scope of the population affected by depression is gradually expanding. Therefore, study of the prevention and treatment of depression is a major issue [5].

In 2001, the World Health Organization (WHO) stated that probiotics, as live micro-organisms, when taken in certain amounts, lead to health benefits for the host [6]. The history of probiotics can be traced to the first use of cheese and fermented products, known to the Greeks and Romans, who recommended their consumption [7]. With further study on the efficacy of probiotics, an increasing number of reports have indicated that they are beneficial to human health and well-being [6]. It has been reported that they can decrease the number of potentially pathogenic gastrointestinal microorganisms and pathogens, and reduce gastrointestinal discomfort, flatulence and bloating, and improve bowel regularity. Probiotics can also enhance the immune system, improve the skin's function, enhance resistance against cedar pollen allergens, and decrease body pathogens, as well as protecting DNA, proteins and lipids from oxidative damage. They can also maintain an individual’s intestinal microbiota in subjects receiving antibiotic treatment [8,9,10,11]. In particular, it has been reported that gut probiotics play a major role in bidirectional communication between the gut and the brain [12,13]. Scientists are increasingly convinced that the vast assemblage of microbiota in our intestines may have a major impact on our state of mind [14]. The effects of gut microbiota on the immune system, brain development, and behavior have attracted attention in recent years. Indeed, gut microbiota may activate the immune and central nervous systems, including commensal and pathogenic microorganisms in the gastrointestinal tract, as gut microorganisms are able to produce and deliver such neuroactive substances as serotonin and gamma-aminobutyric acid [15,16]. Naseribafrouei et al. [17] analyzed fecal samples from 55 individuals (37 patients and 18 non-depressed controls) and found potential correlations between human fecal microbiota (as a proxy for gut microbiota) and depression. Jiang et al. [18] analyzed fecal samples from 46 patients with depression and 30 healthy controls and found increased fecal bacteria in the depressed versus the healthy control group. Experimentally elevated hypothalamic-pituitary-adrenal (HPA) axis responses and depression can be reversed in rats by administering a single bacterium, *Bifidobacterium infantis* [19]. Animal results show that the use of probiotics may lead to an increase in plasma tryptophan levels and reduced concentrations of serotonin in the frontal cortex and of cortical dopamine metabolites, thereby ameliorating depressive symptoms [20]. In another report, the depression scores of rats taking *Lactobacillus rhamnosus* for 28 days declined [21]. Although evidence has indicated that the probiotic combination was not significantly superior to a placebo in relieving symptoms of irritable bowel syndrome (IBS) [22], a meta-analysis performed by Brenner et al. [23] found that *Bifidobacterium infantis 35624* was associated with significant improvement in the composite scores for abdominal pain/discomfort, bloating/distention, and/or bowel movement difficulty compared with a placebo (*p* < 0.05) in two appropriately designed studies. In the double-blind placebo-controlled and randomized parallel group study conducted by Messaoudi et al. [24], healthy volunteers took *Lactobacillus helveticus R0052* and *Bifidobacterium longum* or a placebo for 30 days; the results showed that psychological stress levels, including depression scores, were decreased in subjects who took the probiotics regularly. A study performed by Mohammadi et al. [25] indicated that consuming a probiotic yogurt or a multispecies probiotic capsule for six weeks had beneficial effects on the mental health biomarkers of petrochemical workers. Another study, conducted by Benton et al. [26], found that probiotic yogurt improved the mood of those with an initially poor mood. A study by Akkasheh [27] found that eight weeks of administration of probiotics to patients with major depressive disorder (MDD) had beneficial effects on Beck Depression Inventory scores. The effects of probiotics on human health, especially psychiatric disorders, have recently emerged as an area of interest in neuroscience. Indeed, recent studies have suggested that probiotics have potential effects on mood. As a poor diet has been shown to be a risk factor for depression, a healthy diet would be expected to have a preventive effect on depression [28]. The regulation of probiotics through diet may have critical benefits for preventing and treating depression. To the best of our knowledge, no systematic review of the effects of probiotics on depression has ever been published. Thus, we conducted a systematic review of randomized controlled trials (RCTs) in an attempt to summarize the evidence on the relationship between probiotics and depression and to identify heterogeneity among the RCT results.

## 2. Methods

### 2.1. Inclusion Criteria

Studies were deemed eligible if they met the following inclusion criteria: (1) described a randomized controlled trial (RCT); (2) included a clinical cohort and controls, and the clinical cohort’s intervention was the consumption of probiotics; (3) reports used similar methods and scientific rating scales for depression; and (4) the scales were reported as mean ± SDs. When the same groups of patients were reported in multiple papers, only the most recent and complete paper was selected to avoid overlap.

### 2.2. Exclusion Criteria

Studies that had the following traits were excluded: (1) there was no control group in the study; (2) publications contained findings that had already been published; (3) results were not described as means ± SDs; and (4) the study did not meet our search inclusion criteria.

### 2.3. Search Strategy

Two reviewers searched databases and other sources including PubMed, Medline, Springer, Elsevier Science, EMBASE, Cochrane Library, China Knowledge Resource Integrated (CNKI) from the earliest record of the databases to 1 January 2016, using the search terms “probiotics”, “prebiotics”, “Lactobacillus”, “Bifidobacterium”, “saccharomyces”, “depression” or “mental health”. References from these publications were also reviewed to identify additional studies. Publications were limited to the English language, and literature that only had conference abstracts were excluded due to the lack of sufficient data.

### 2.4. Data Collection

Data relating to the effects of probiotics on depression were extracted using a tailored form and checked by the second reviewer. The form included study demographics, trial design, probiotic regimens and outcomes (Table 1). If the study data were unclear, we corresponded with the author to obtain further information.

### 2.5. Statistical Analysis

The analysis was performed with RevMan 5.3 software (the Cochrane Collaboration, 2014, Nordic Cochrane Center, Copenhagen, Denmark). Continuous outcome variables are expressed as mean differences (MDs), 95% confidence intervals (CIs) were analyzed as summary statistics, and a fixed-effects model was used based on the heterogeneity of outcomes across studies. Where outcome measures were comparable, the datasets were pooled in the meta-analysis. *I*^2^ was used to evaluate between-study heterogeneity. Statistical heterogeneity was checked using the χ^2^ test, and the extent of inconsistency was assessed by the *I*^2^ statistic. Both the fixed-effects model and the random-effects model were considered in the analysis depending on the *I*^2^ result. If the *I*^2^ ≥ 50%, the result was considered to have significant heterogeneity, and a random-effects model was used to calculate the parameters. Conversely, in the absence of heterogeneity, a fixed-effects model was assumed. The funnel plot was used to test if there was publication bias when the datasets contained at least three studies. A two-tailed *p*-value of < 0.05 was considered statistically significant. A sensitivity analysis was performed by excluding studies one by one or by excluding studies involving a group of subjects with the same disease. We also conducted a subgroup analysis based on age and depression status. We also analyzed the risk of bias for each included RCT for the efficacy of probiotics. The criteria were judged according to the Cochrane “risk of risk” assessment tool.

## 3. Results

### 3.1. Included Studies

**Figure 1:**
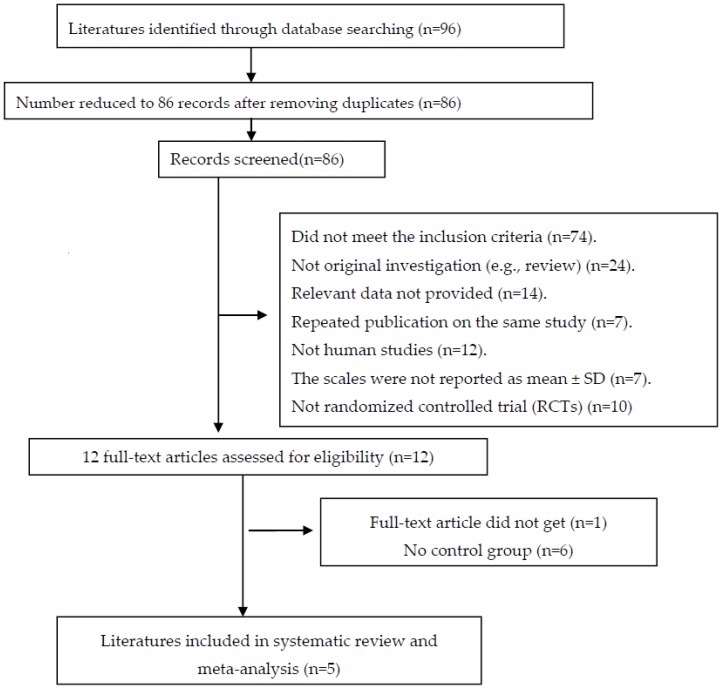
An adapted PRISMA flow diagram.

An adapted PRISMA flow diagram [29] shows the process followed to select the papers used in this report (Figure 1). In total, 96 publications were reviewed. Finally, five clinical trials [24,25,27,30,31] (involving 183 cases and 182 controls) were incorporated based on the inclusion and exclusion criteria in the pooled analysis. The characteristics of the studies are shown in Table 1.

### 3.2. Quality Assessment

**Figure 2:**
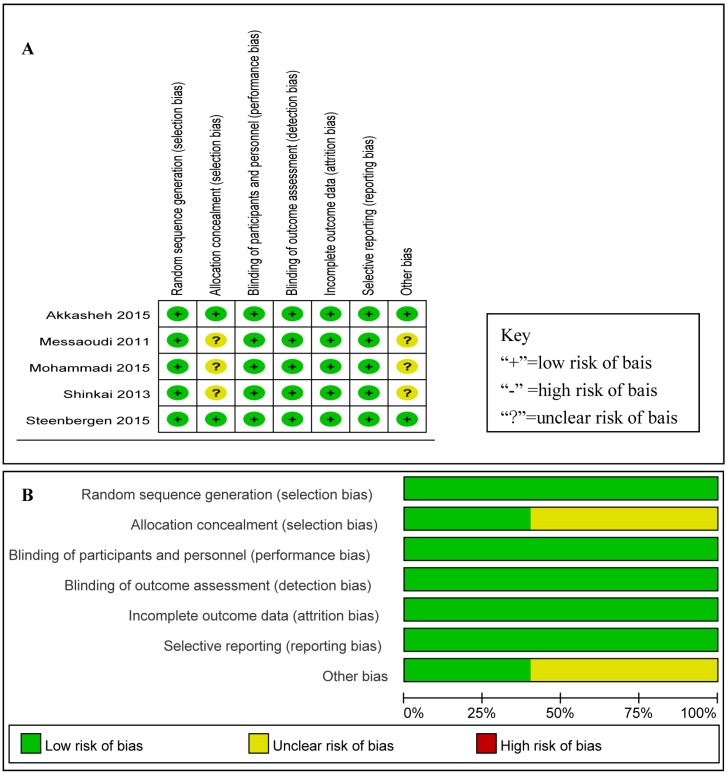
(**A**) risk of bias for each RCT included: low risk of bias (+), high risk of bias (-), unclear risk of bias (?); and (**B**) bar chart comparing the percent risk of bias for each RCT included. The figure shows that the risk of bias was quite low.

**Figure 3:**
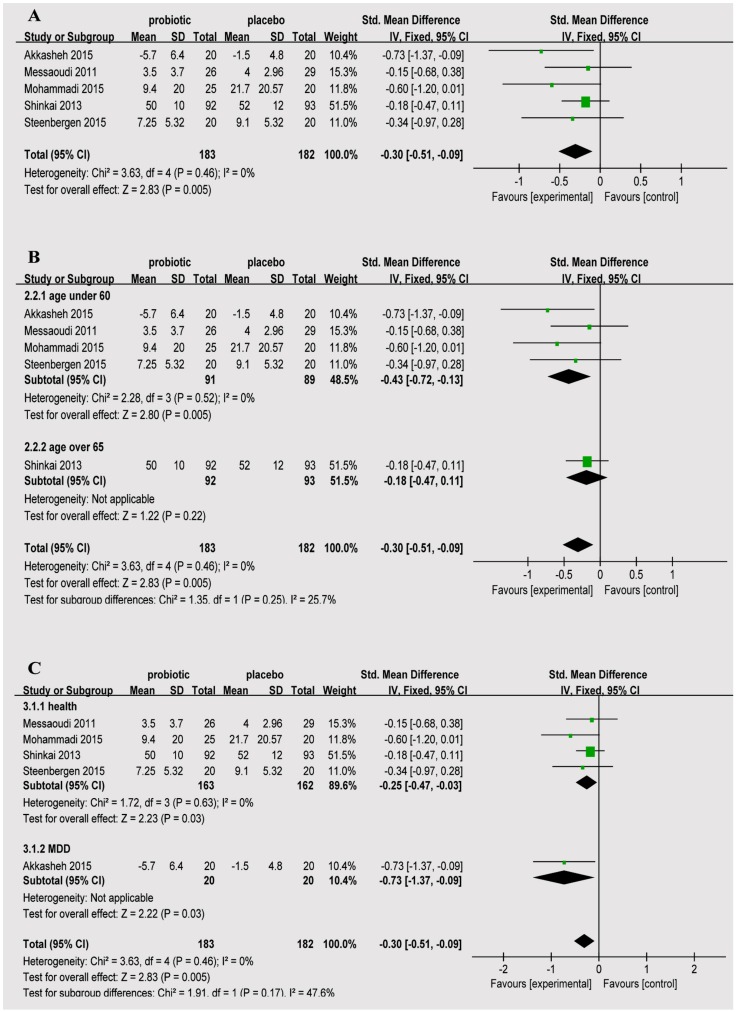
(**A**) estimates for probiotics associated with depression in the meta-analysis; (**B**) forest plots for different ages; and (**C**) forest plots for depression status.

Risk of bias for efficacy analysis for each included RCT is shown in Figure 2A and Figure 3B shows the risk of bias across all RCTs. These data show that the highest overall risk of bias was in relation to performance and detection. According to the quality assessment of studies, the five studies included were all RCTs, and the risk of bias for each RCT included was low.

Three studies divided the subjects into two groups (probiotic, control); one study’s subjects were randomly divided into three groups to receive 100 g/day probiotic yogurt plus one placebo capsule or one probiotic capsule daily plus 100 g/day conventional yogurt or 100 g/day conventional yogurt plus one placebo capsule. Considering the comparability between the studies, one probiotic capsule daily plus 100 g/day conventional yogurt and 100 g/day conventional yogurt plus one placebo capsule were analyzed. Another study divided subjects into a high-dose group, a low-dose group and a control group, so we selected the low-dose group whose dose was closer to the initial study. There were four double-blind studies and one triple-blind study; all five studies reported baseline data of each group; and the differences between the baseline and the groups were not statistically significant.

### 3.3. Efficacy of Probiotics

As shown in Figure 3, the meta-analysis comparing the probiotic to the control group showed MD = −0.30 (95% CI: −0.51–−0.09), *p* = 0.005.

A subgroup analysis was performed to investigate age with depression status to observe the relationship between probiotics and depression scale (Figure 3B,C). The ages of the subjects in four of the studies were 60 years and below, MD = −0.43 (95% CI: −0.72–−0.13), *p* = 0.005; one study’s subjects’ were 65 years and older, MD = −0.18 (95% CI: −0.47–−0.11), *p* = 0.22. The subjects in four studies were healthy individuals without depression, MD = −0.25 (95% CI: −0.47–−0.03), *p* = 0.03; one study’s subjects were patients with major depression, MD = −0.73 (95% CI: −1.37–−0.09), *p* = 0.03.

The stability of the results was tested by sensitivity analysis. We sequentially removed a study in all of the above analyses that did not reach significance, suggesting that the results of our meta-analysis were not significantly unstable.

### 3.4. Publication Bias

**Figure 4:**
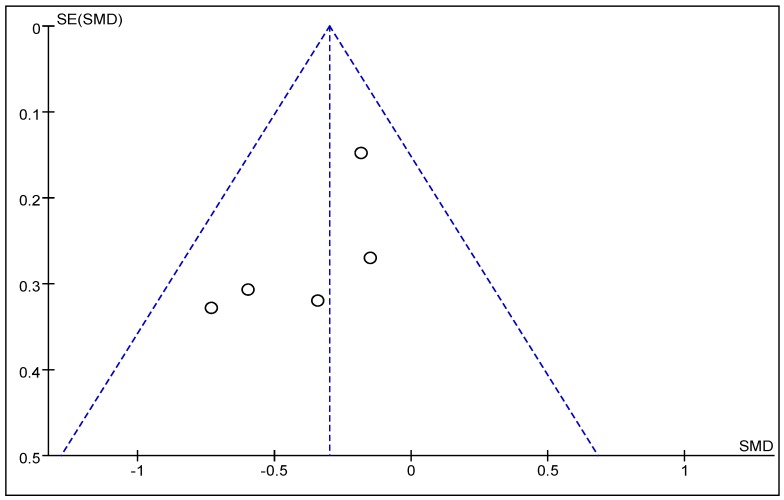
Funnel plot analysis. There was low heterogeneity among the studies, and no evidence of publication bias.

A funnel plot was used to qualitatively assess for publication bias. The funnel plot shown in Figure 4 is partially symmetrical, indicating no obvious evidence of asymmetry, and, therefore, no evidence of publication bias.

## 4. Discussion

Major depressive disorder, often called simply “depression,” refers to a psychological state characterized by a “low” mood and an aversion to activity; this state that can affect cognition, behaviors, feelings, and one’s overall sense of well-being [32]. In the United States, approximately 3.4% of people with major depression die by suicide, and up to 60% of people who die by suicide suffered from depression or another mood disorder [33,34]. Moreover, this syndrome is being diagnosed more frequently in developed countries, where it affects up to 20% of the population at some stage of their lives [35,36]. According to the World Health Organization (WHO), depression is currently the fourth leading cause of the global burden of disease; it is predicted that depression will be ranked second by 2020. The WHO has predicted that, by 2030, depression will account for the highest level of disability attributable to any physical or mental disorder worldwide [37]. Many treatment strategies have been used to fight this disease, including pharmaceuticals such as selective serotonin reuptake inhibitors (SSRIs) [38] and lithium [39]; medical technologies such as electroconvulsive therapy [40], deep brain stimulation, and bright light therapy, exercise, and music therapy. Medication appears to be effective, but its effect may be significant in only the most severely depressed individuals. Depressed individuals have shorter life expectancies than those without depression, in part because of their greater susceptibility to medical illnesses and suicide. It is unclear whether medications affect the risk of suicide.

The ongoing exploration of the human microbiome promises to clarify the link between the gut and the brain [14]. Scientists are increasingly recognizing that the gut microbiome might influence neuropsychiatric symptoms and might be a tractable target for novel treatment options [28]. A study conducted by Cryan [41] showed that disruption of the microbiome induced mice to behave in ways that mimicked human anxiety, depression, and even autism. Thus, as Cryan [41] noted, “that dietary treatments could be used as either [an] adjunct or sole therapy for mood disorders is not beyond the realm of possibility”. Cryan [42] has reported that two varieties of Bifidobacterium were more effective than Lexapro (Discovery Fine Chemicals, Dorset, UK), an agent used to treat anxious and depressed behaviors, in a laboratory mouse strain.

A study conducted by Desbonnet [19] assessed the potential benefits of the probiotic *Bifidobacterium infantis* using the rat maternal separation (MS) model, a paradigm that has proven to be of value in the study of stress-related gastrointestinal (GI) and mood disorders. Probiotic treatment resulted in normalization of the immune response and reversal of behavioral deficits. Desbonnet [19] also found attenuation of pro-inflammatory immune responses and a rise in tryptophan, a serotonergic precursor, due to *Bifidobacterium* treatment; this study provides encouraging evidence in support of the proposition that this probiotic may possess antidepressant properties. Gilbert [2] indicated that a high-PUFA n-3 diet or the administration of probiotics, starting after the onset of reperfusion, helped attenuate apoptosis in the limbic system and post-MI depression in rats. In a double-blind placebo-controlled and randomized parallel group study by Messaoudi et al. [24], healthy volunteers took *Lactobacillus helveticus R0052* and *Bifidobacterium longum* or a placebo for 30 days; the results showed decreased psychological stress, including depression, in subjects who took the probiotics regularly. A study by Mohammadi et al. [16] indicated that consuming a probiotic yogurt or a multispecies probiotic capsule for six weeks had beneficial effects on the mental health biomarkers of petrochemical workers [25]. Another study, conducted by Benton et al. [26], found that probiotic yogurt improved the mood of those with an initially poor mood.

In the present study, we investigated the effects of probiotics on depression. One of the five studies included examined individuals with major depression; the remaining four studies examined non-depressed individuals. The findings suggest an important role for probiotics in reducing the risk of depression in non-depressed individuals. The studies were heterogeneous in strain, dose and duration of probiotics. The subjects’ ages, depressive state and therapies received varied with each study. In addition, the outcomes assessed varied, potentially explaining some of the between-study heterogeneity of the results. The risk of bias on the effect of the RCTs was low according to the analysis. Sensitivity analyses showed no qualitative changes in conclusions where meta-analyses were still possible, when the differences between studies were assessed. The meta-analysis found that those in the probiotic group had a significantly reduced incidence of depression, MD = −0.30 (95% CI: −0.51–−0.09), *p* = 0.005. Most of the individual studies did not report significant results. The study conducted by Messaoudi [24] showed MD = −0.15 (95% CI: −0.68–0.38); the one conducted by Mohammadi [25] showed MD = −0.60 (95% CI: −1.20–0.01); that conducted by Shinkai [31] showed MD = −0.18 (95% CI: −0.47–0.11); and that conducted by Steenbergen [30] showed MD = −0.14 (95% CI: −0.97–0.28). Only the study performed by Akkasheh [27] showed MD = −0.73 (95% CI: −1.37–−0.09). These results can be explained by our meta-analysis. The basic tenet of a meta-analysis is that there is a common truth behind all conceptually similar scientific studies; however, individual studies contain a certain degree of error. Thus, the aim of a meta-analysis is to use statistical approaches to derive a pooled estimate that is closest to the unknown common truth based on how this error is perceived. The advantage of this approach is that the aggregation of information leads to a higher level of statistical power and more robust point estimates than would be possible based on the measures in any individual study.

The subgroup analysis of age showed that for subjects aged 60 and below, oral probiotics can effectively reduce depression rating scales, and that, for people aged 65 and older, no effect was observed, indicating that the probiotic antidepressive effects were different in different age groups; however, because there was only one study with subjects aged 65 years and above, it is impossible to draw any strong conclusion.

The subgroup analysis of different depressive states showed that the administration of probiotics to patients with depression and to healthy volunteers could effectively reduce depression rating scales, which is unlike some of the conclusions in which improvements in mood after probiotics administration only occurred in participants who showed elevated symptoms of depression at baseline. This suggests that non-depressed people may reduce the risk of depression by receiving oral probiotics.

Although comprehensive and complete document retrieval was applied in this meta-analysis to decrease publication bias, and each step of meta-analysis was performed by two separate researchers to reduce the deviation of analysis, this study has some limitations. First, the probiotics selected in the reference documents, and the dose and treatment, are not the same, and other interferences such as diet and medication could also have affected the result. In addition, the reference documents are RCTs that are from different countries; thus, people with different genetic constitutions or microbial exposure may have a different response to identical probiotics. Finally, the depression rating scales chosen by the research projects are different, and this may have affected the meta-analysis. Finally, some of the included studies had a small sample size, which might have influenced the reliability and validity of the conclusions.

## 5. Conclusions

This systematic review supports the potential role of probiotics in reducing the risk of depression. Further evidence from larger samples and more rigorous RCTs are needed to determine whether probiotics can significantly reduce the overall risk of depression.

## Figures and Tables

**Table 1:** Characteristics of included RCTs for meta-analysis.

Study, Year, Country	Subjects, Total Number of Cases	Take Medications; Duration	Species, Dosage	Scale of Depression
Mohammadi, 2015, Iran	20–60 years old healthy petrochemical workers, 45 (20/25)	6 weeks	*Actobacillus casei* 3 × 10^3^ CFU/g, *L. acidophilus* 3 × 10^7^ CFU/g, *L. rhamnosus* 7 × 10^9^ CFU/g, *L. bulgaricus* 5 × 10^8^ CFU/g, *Bifidobacterium breve* 2 × 10^10^ CFU/g, *B. longum* 1 × 10^9^ CFU/g, *S. thermophilus* 3 × 10^8^ CFU/g.	Depression Anxiety and Stress Scale (DASS)
Akkasheh, 2015, Iran	20–55 years old patients with MDD, 40 (20/20)	One capsule per day; 8 weeks	*L. acidophilus* 2 × 10^9^ CFU/g, *L. casei* 2 × 10^9^ CFU/g, *Bifidobacterium bifidum* 2 × 10^9^ CFU/g.	Beck Depression Inventory (BDI)
Steenbergen, 2015, The Netherlands	Around 20 years old; healthy young adults, 40 (20/20)	One sachet per day; 4 weeks	*Bifidobacterium bifidum W23*, *Bifidobacterium lactis W52*, *L. acidophilus W37*, *L. brevis W63*, *L. casei W56*, *L. salivarius W24*, *Lactococcus lactis* (*W19* and *W58*), 2.5 × 10^9^ CFU/g.	BDI
Messaoudi, 2011, France	30–60 years old healthy human volunteers, 55 (29/26)	1.5 g/day; 30 days	*Lactobacillus helveticus R0052*, *Bifidobacterium longum R0175*, 3 × 10^9^ CFU.	Hospital Anxiety and Depression Scale (HADS-D)
Shinkai, 2013, Japan	Adults aged 65 years or older, 278 (93/92/93)	One capsule per day, 20 weeks	*Lactobacillus pentosus strain b240*, *Low-dose group* 2 × 10^9^ cells, *High-dose group* 2 × 10^10^ cells.	Profile of Mood States (POMS): Depression-dejection
